# Regional disparities in interferon therapy for chronic hepatitis C in Japan: a nationwide retrospective cohort study

**DOI:** 10.1186/s12889-015-1891-2

**Published:** 2015-06-19

**Authors:** Naohiko Masaki, Yoko Yamagiwa, Takuro Shimbo, Kazumoto Murata, Masaaki Korenaga, Tatsuya Kanto, Masashi Mizokami, and the prefectural members contributing to the Japanese Interferon Database

**Affiliations:** The Research Center for Hepatitis and Immunology, National Center for Global Health and Medicine, 1-7-1 Kohnodai, Ichikawa, Chiba 272-8516 Japan; Department of Clinical Study and Informatics, Center for Clinical Sciences, National Center for Global Health and Medicine, Tokyo, 162-8655 Japan

**Keywords:** Treatment performance, Treatment outcome, Peginterferon-α, Ribavirin, Subsidy policy

## Abstract

**Background:**

Many patients with chronic hepatitis C have been treated with interferon (IFN) therapy in Japan, especially after the introduction of subsidies for medical expenses in 2008. However, its performance and outcome have never been evaluated. Therefore, a nationwide, mail-based, retrospective cohort study was conducted.

**Methods:**

Regional disparities in the demographic features, treatment performance, and virological response were evaluated using an intent-to-treat design. The participating prefectures were classified into nine regions from north to south (Hokkaido/Tohoku, Kanto, Shin-etsu, Hokuriku, Tokai, Kinki, Chugoku, Shikoku, and Kyushu). Multivariate logistic regression analysis was performed to select predictive factors for treatment performance and outcome.

**Results:**

From December 2009 to May 2013, 16,854 patients with chronic hepatitis C were registered from 37 prefectures in Japan (median age: 60 years; 50.4 % male; 74.8 % IFN-naïve; HCV genotype [1 or 2]/viral load [high (≥5 log IU/mL) or low (<5 log IU/mL)]: 1/high = 58.2 %, 1/low = 5.2 %, 2/high = 27.3 %, 2/low = 7.5 %; 83.4 % treated with peginterferon-α and ribavirin). Mean age, proportion of elderly patients (≥65 years), male sex, IFN-experienced, and HCV genotype were significantly different among the nine regions (all *P* < 0.001). Regional disparities were independently selected as one of the predictive factors for treatment performance and outcome in patients treated with peginterferon-α and ribavirin, which revealed two regions that required further investigation.

**Conclusions:**

Regional disparities still exist in IFN therapy, and are strongly associated with treatment performance and outcome. Since the accessibility to medical resources for individual patients seemed to be different among the nine regions, public health actions should be focused on how to construct and properly manage consultation networks between base hospitals and local clinics, especially in those regions with low population density.

**Electronic supplementary material:**

The online version of this article (doi:10.1186/s12889-015-1891-2) contains supplementary material, which is available to authorized users.

## Background

There are 130–150 million people infected with hepatitis C virus (HCV) worldwide, and 350,000–500,000 patients die of HCV-related liver diseases annually (e.g., liver cirrhosis or hepatocellular carcinoma) [1]. Standard treatment for chronic hepatitis C (CHC) has been peginterferon-α and ribavirin (P/R), and the sustained virological response (SVR) rate has remained at 50 %, in difficult-to-treat cases of HCV genotype 1 and high viral load [2]. The introduction of protease inhibitors, such as boceprevir [3, 4], telaprevir [5, 6], or simeprevir [7, 8] could improve the SVR rate up to 75–85 % in interferon (IFN)-naïve cases. Furthermore, the era of IFN-free treatment with oral-only directly acting antivirals (DAAs) has just become a reality with SVR rate > 90 % [9–11].

Standardized performance and outcome of antiviral therapy are essential to eradicating HCV, which could significantly decrease the risk of progression to liver diseases (e.g., liver cirrhosis and hepatocellular carcinoma). For this purpose, the Japanese government and 47 local governments started nationwide strategies from January 2007, to build intensive treatment networks in all prefectures, authorized later by the Basic Act on Hepatitis Measures (Act No. 97, December 4, 2009) [12]. Almost concurrently, subsidies for antiviral treatment (e.g., IFN therapy for patients infected with hepatitis B virus [HBV] or HCV, or nucleoside analogs for those infected with HBV) were introduced to provide more patients with a higher chance of virological response. For example, the total medical expenses for 48 weeks of standard care for a patient with CHC could reach 23,930 USD for peginterferon-α2a (180 μg = 278.3 USD) and ribavirin (800 mg = 31.5 USD) combination therapy. The patient will have to pay 600 USD for P/R every month, as the average coverage of health insurance is 70 % in Japan. With the aid of this subsidy policy, the patients will only have to pay ~100–200 USD monthly according to their taxable income. More than 117,000 patients benefited from this subsidy policy during the initial 4 years (April 2008 to March 2012). The fact that the substantial amount of public money was allocated for the subsidy policy urged us to perform a nationwide retrospective study to evaluate whether the performance and outcome of IFN therapy have been standardized throughout Japan.

## Methods

### The outlines of the study

The Hepatitis Information Center of the National Center for Global Health and Medicine (Chiba, Japan) started a retrospective cohort study to construct the Japanese Interferon Database in December 2009. All 47 prefectural governments were invited to join this project. Currently, 37 prefectures have been participating and sending data to the Hepatitis Information Center. The standard duration of P/R therapy was 48 weeks and 24 weeks for genotype 1 and non-genotype 1, respectively, and the final therapeutic outcome was determined 24 weeks after the treatment period, according to the guidelines of the American Association for the Study of Liver Diseases [13] and the Japan Society of Hepatology [14]. The local governments had the application forms submitted by each patient, in which the genotype or serotype of HCV, viral load, scheduled date of treatment, and demographic features of the patients were described. Therefore, the requests to draw up the reports on therapeutic outcomes were made in a timely manner to the relevant doctors by the local governments.

Most of the enrolled patients were treated by P/R with weekly administration of peginterferon-α2a (Pegasys; Chugai Pharmaceutical, Tokyo, Japan) and daily ribavirin (Copegus; Chugai Pharmaceutical), or weekly peginterferon-α2b (Pegintron; MSD, Tokyo, Japan) and daily ribavirin (Rebetol; MSD). The dose of peginterferon-α2a, regardless of the patient’s body weight, was 180 μg. However, the dose of peginterferon-α2b was adjusted based on the patient’s body weight as follows: patients weighing ≤45 kg, >45 kg and ≤60 kg, >60 kg and ≤75 kg, >75 kg and ≤90 kg, and >90 kg were given 60 μg, 80 μg, 100 μg, 120 μg, and 150 μg of peginterferon-α2b weekly, respectively. Patients weighing ≤60 kg, >60 kg and ≤80 kg, and >80 kg were given 600 mg, 800 mg, and 1000 mg of ribavirin daily, respectively. Dose modification of peginterferon-α or ribavirin was based on the manufacturers’ recommendations.

The format of the reports was unified and the demographic features of the patients included sex, date of birth, scheduled treatment period, previous history of IFN therapy, clinical and/or histological diagnosis, and IFN regimen. The data for virological markers, including viral load and serum transaminases levels, were collected before treatment, at cessation of treatment, and at final analysis. Peripheral platelet counts during the pre-treatment period were included. Determination of serotype and/or genotype (if possible) was a prerequisite for the standard treatment of chronic HCV infections in Japan. HCV RNA was determined by quantitative real-time polymerase chain reaction (COBAS AmpliPrep/COBAS TaqMan HCV Test; Roche Molecular Systems, Pleasanton, CA, USA). A high viral load was considered ≥5.0 log10 IU/mL HCV RNA. Together with these demographic features, information regarding virological outcome, treatment performance (i.e., accomplishment or withdrawal), reasons for treatment withdrawal (i.e., severe adverse events or other unrelated events), and personal information including patient identification and written informed consent were reported to the local governments. Thereafter, the data without personal information were sent to the Hepatitis Information Center with assigned temporary identifications to maintain anonymity in future references.

**Fig. 1:**
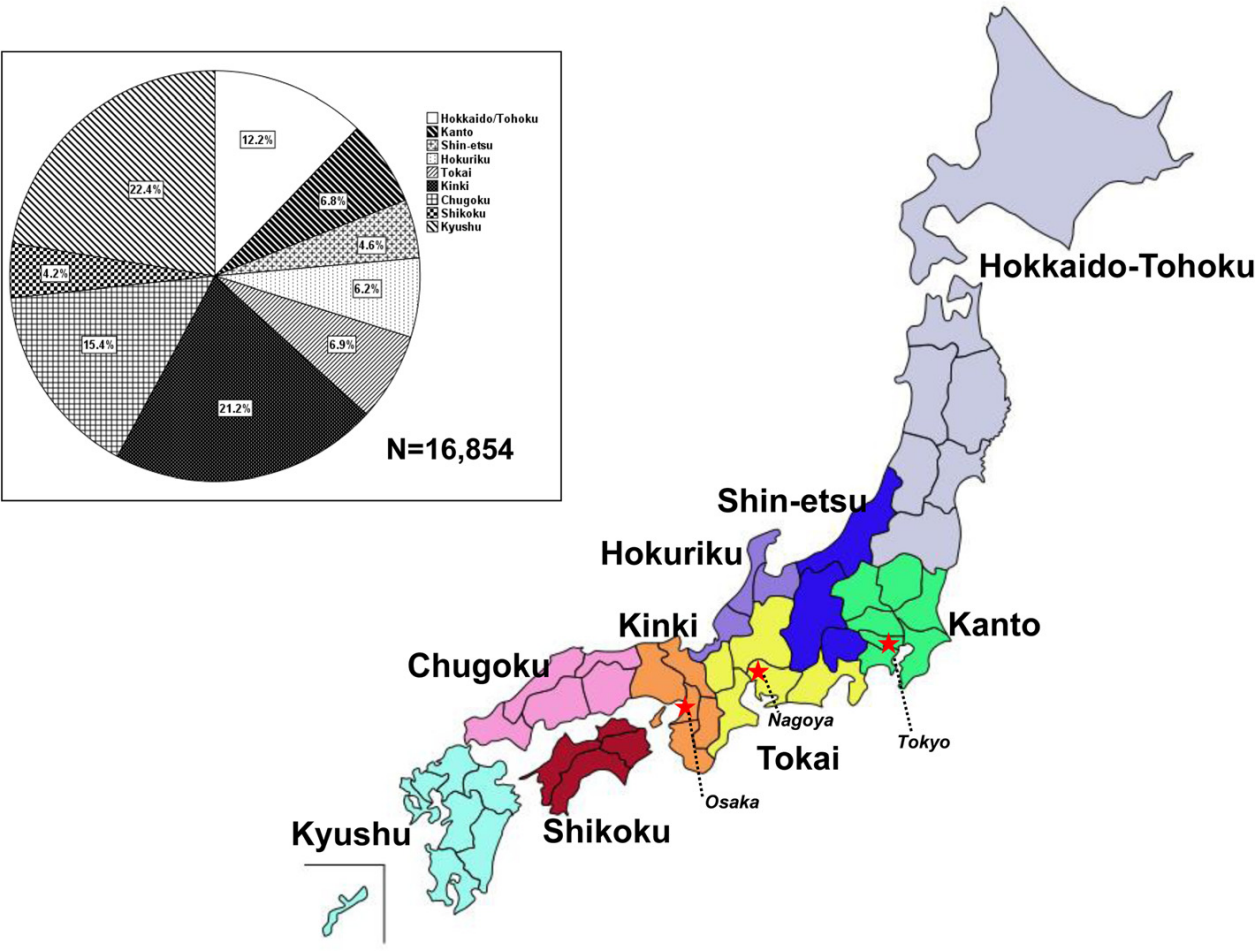
A map showing nine regions of Japan. The percentages of CHC cases for each region are shown in the small inset. The three biggest cities, Tokyo, Osaka and Nagoya are located in Kanto, Kinki and Tokai, respectively

To analyze possible regional disparities in treatment performance and virological response, the 37 prefectures participating in this study were classified into nine regions from north to south as follows (numbers in parentheses denote the number of participating prefectures in each region): Hokkaido/Tohoku (6), Kanto (4), Shin-etsu (3), Hokuriku (3), Tokai (3), Kinki (6), Chugoku (4), Shikoku (3), Kyushu (5) (Fig. 1).

### Virological response

Virological response was assessed by each doctor in charge according to the standard criteria described below. SVR was defined as undetectable HCV RNA levels in serum 24 weeks after cessation of treatment, while transient virological response (TVR) was defined as reappearance of HCV RNA in serum following undetectable HCV RNA at cessation of treatment. Nonvirological response (NVR) was defined as <2 log-unit decline in serum HCV RNA levels from the pre-treatment baseline value within the first 12 weeks, or detectable viremia at 24 weeks after treatment initiation. Patients who were withdrawn from treatment because of the presence of serum HCV RNA at 24 weeks of therapy, or viral breakthrough, or who were lost during treatment or follow-up were included in the intent-to-treat analysis.

### Evaluation of liver fibrosis

Since liver biopsy has not been regularly performed in recent clinical settings, a simple non-invasive index (Fibrosis-4 [FIB-4] index), which correlates well with hepatic fibrosis as determined by liver biopsy [15], was used to evaluate the extent of liver fibrosis. The FIB-4 index was used for multivariate logistic regression analysis, instead of clinical diagnoses.

### Ethics statement

The study protocol complied with the Helsinki Declaration and was approved by the Ethics Committee of the National Center for Global Health and Medicine, Japan (#738; October 1, 2009). Written informed consent was obtained from the patients prior to enrollment.

### Statistical analysis

Continuous variables were expressed as median and interquartile ranges, unless otherwise specified, and compared using the Mann–Whitney *U* test or Kruskal–Wallis analysis. Categorical variables were compared using Pearson’s *χ*^2^ test or Fisher’s exact test. The demographic features of the patients were compared among the nine regions using Kruskal–Wallis analysis or Pearson’s *χ*^2^ test. Multivariate analysis was performed using a simultaneous, non-stepwise, logistic regression analysis, with all examined parameters, regardless of the univariate analysis results. All *P*-values were two-tailed, and *P* < 0.05 was considered statistically significant. Data analyses were performed using IBM SPSS Statistics for Windows, Version 20.0 (IBM Corp., Armonk, NY, USA).

## Results

### Patient demographics

From December 2009 to May 2013, 17,169 reports were sent to the Hepatitis Information Center. The etiology of chronic liver diseases was HBV alone (*n* = 315), HCV alone (*n* = 16,838), and co-infection with HBV and HCV (*n* = 16). Hence, 98.2 % of the reports (*n* = 16,854) were HCV related and further analyses were confined to patients with HCV infection. The percentages of reports from each region are shown in the small inset of Fig. 1. The age distributions of this cohort at every decade were as follows: <9 years (*n* = 3); 10–19 years (*n* = 24); 20–29 years (*n* = 310); 30–39 years (*n* = 831); 40–49 years (*n* = 2141); 50–59 years (*n* = 4916); 60–69 years (*n* = 6625); 70–79 years (*n* = 1915); >80 years (*n* = 45); and unknown (*n* = 44). The median age was 60.0 years (interquartile range, 52.0–66.0 years). Demographic features (i.e., mean age, proportion of elderly patients [≥65 years], male sex, IFN treatment, and HCV genotype distribution) differed significantly among the nine regions (*P* < 0.001; Table 1).

**Table 1:** Demographic features of patients with chronic hepatitis C treated with interferon (IFN) in nine regions of Japan

	All	Hokkaido/Tohoku	Kanto	Shin-etsu	Hokuriku	Tokai	Kinki	Chugoku	Shikoku	Kyushu	*P*-value
n	16854	2055	1142	781	1046	1170	3565	2599	716	3780	
Age (years)^a^	57.9 ± 10.9	56.9 ± 10.5	56.5 ± 11.3	59.4 ± 10.1	60.2 ± 9.3	58.7 ± 10.4	57.3 ± 11.6	59.3 ± 10.7	57.8 ± 10.1	57.5 ± 11.1	<0.001^b^
The elderly (≥65 years) (%)	30.2	26.1	24.9	34.4	35.3	32.5	29.9	34.9	26.7	28.7	<0.001^c^
Gender male (%)	50.4	50.6	53.3	46.7	44.3	48.8	51.5	49.7	54.6	51.2	<0.001^c^
IFN-experienced cases (%)	25.2	25.4	18.8	26.1	28.7	22.3	23.7	28.8	31.2	24.4	<0.001^c^
HCV Genotype 1/2/3/undetermined (%)	63.5/34.7/ 0.2/1.7	64.2/32.8/ 0.0/2.9	56.4/42.0/ 0.1/1.5	68.5/29.4/ 0.0/2.1	67.3/30.9/0.0/1.8	63.1/35.1/ 0.2/1.6	65.0/32.1/ 0.4/2.5	64.2/34.9/ 0.4/0.5	56.7/41.9/ 0.1/1.3	62.5/36.4/ 0.0/1.0	<0.001^c^

^a^Age is shown as mean ± standard deviation

^b^Kruskal–Wallis analysis

^c^Pearson’s *χ* ^2^ test

*IFN*, Interferon; *HCV*, Hepatitis C virus

### Treatment performance and outcome in patients treated by peginterferon-α and ribavirin

**Fig. 2:**
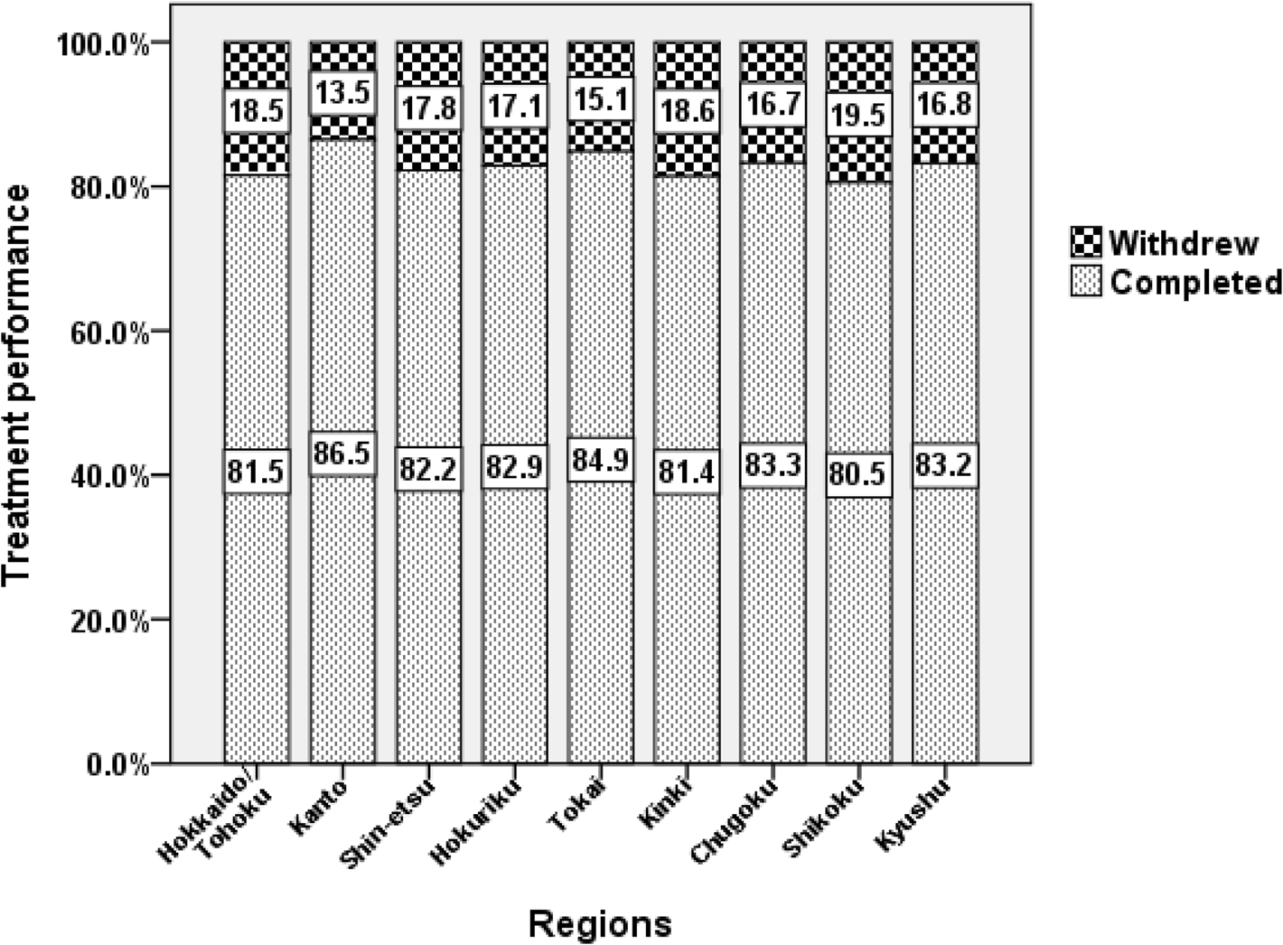
Regional disparities in treatment performance in patients with chronic hepatitis C treated by peginterferon-α and ribavirin (P/R). As for the rates of treatment accomplishment, the average of the P/R-treated cohort was 82.8 %, and was higher than the average in five regions (Kanto, Hokuriku, Tokai, Chugoku, and Kyushu), and lower than average in the other four. The rates of treatment accomplishment differed significantly among the nine regions (*P* = 0.009)

**Fig. 3:**
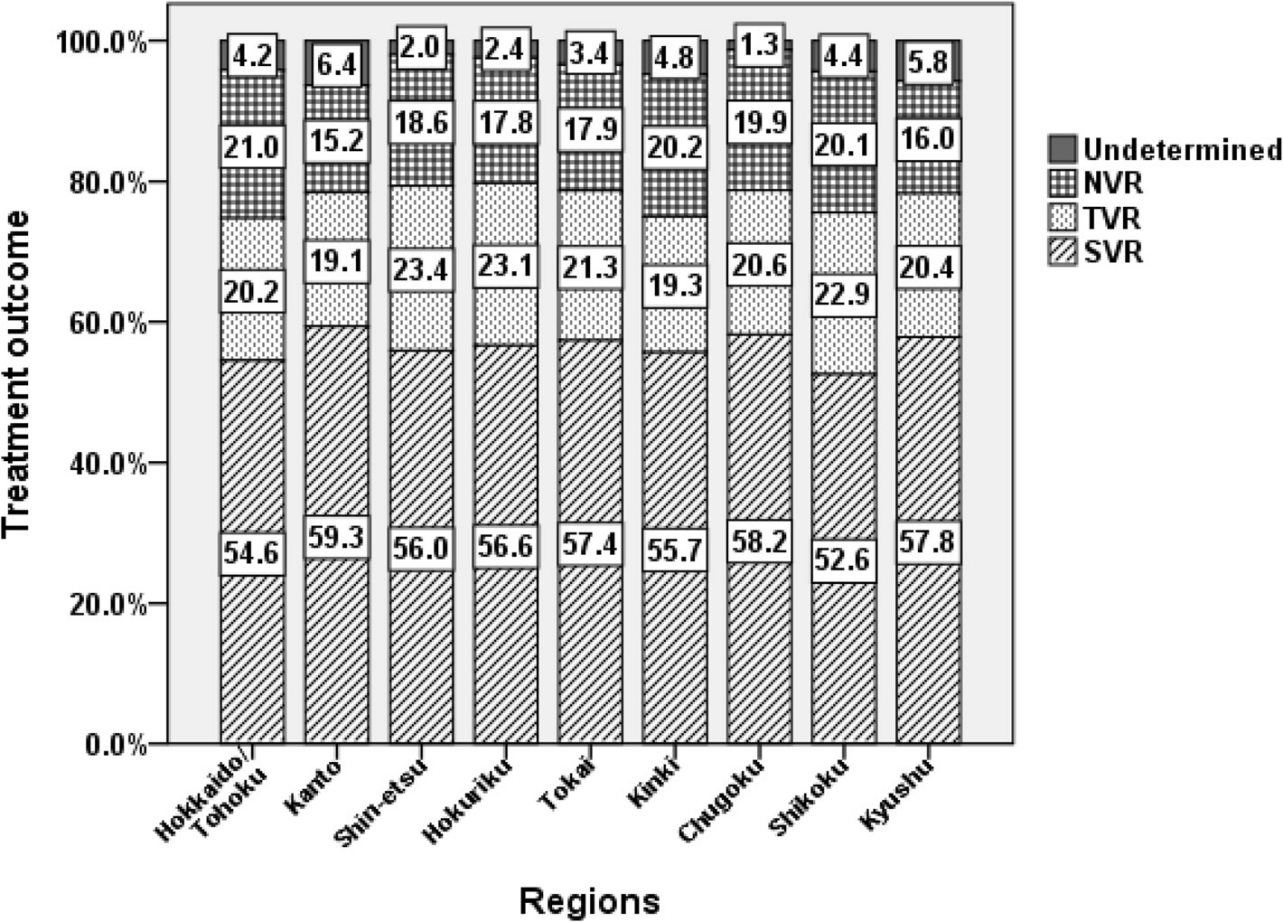
Regional disparities in treatment outcome in patients with chronic hepatitis C treated by peginterferon-α and ribavirin (P/R). In the entire P/R-treated cohort (*n* = 14,061), the treatment outcome was as follows: the rates of sustained virological response (SVR), transient virological response (TVR), non-virological response (NVR), and undetermined response were 56.7 %, 20.6 %, 18.6 %, and 4.1 %, respectively. There were regional disparities among the nine regions in treatment outcome (*P* < 0.001)

**Fig. 4:**
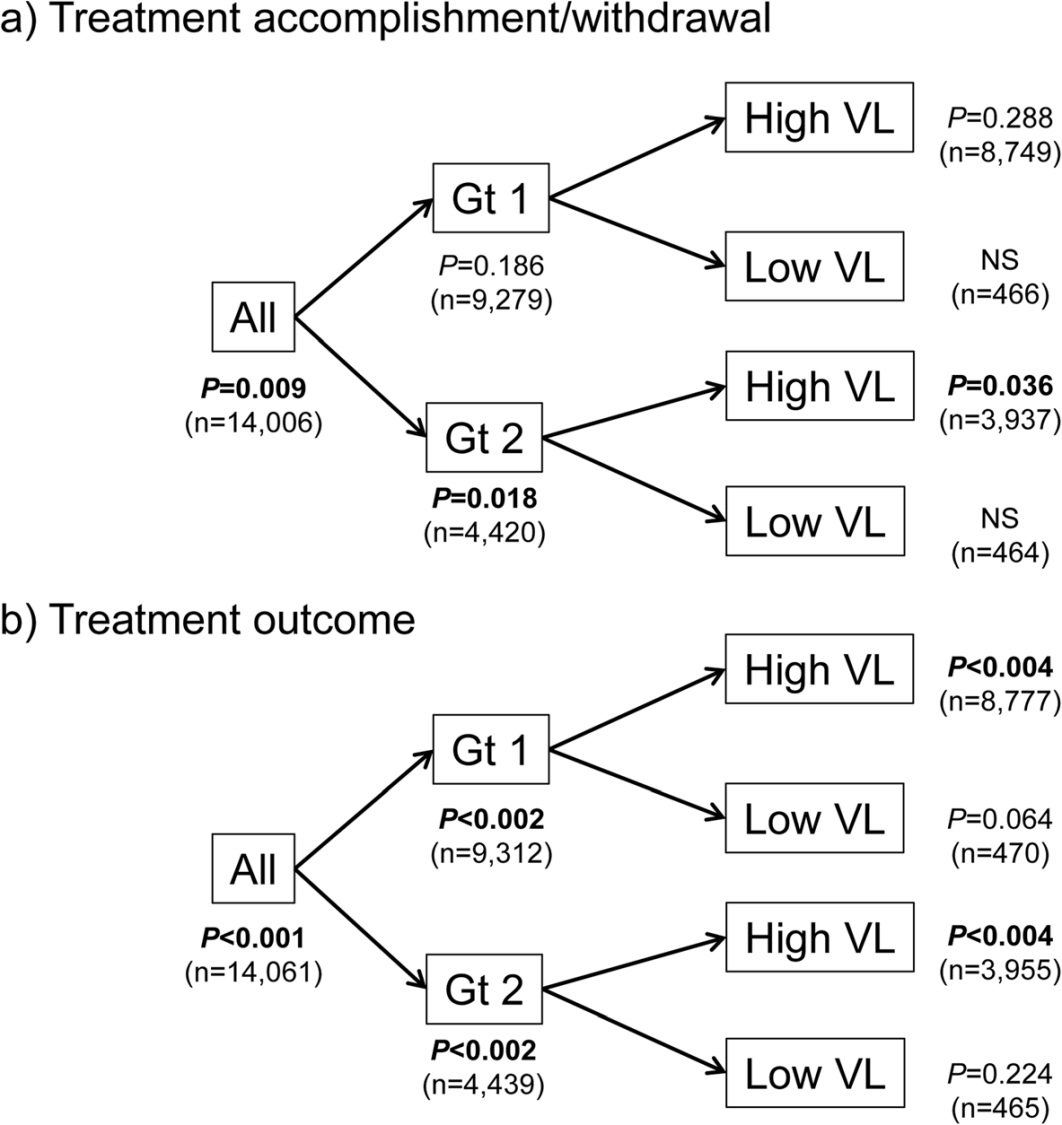
Summary of regional disparities in treatment performance and outcome, in subgroups stratified with genotypes and viral loads of hepatitis C virus (HCV). **a** Treatment performance. Regional disparities were detected in the genotype 2 subgroup (*P* = 0.018), especially with high viral load (*P* = 0.036), but not in genotype 1. **b** Treatment outcome. Regional disparities were confirmed in the subgroups with high viral load, regardless of HCV genotypes. The *P-*values were adjusted with the Bonferroni method for the number of strata

About 83.4 % (*n* = 14,061) of the patients were treated with P/R. Therefore, further analyses were restricted to this regimen to clarify the current treatment performance and outcome in Japan. The average percentage of treatment accomplishment in the P/R-treated cohort was 82.8 %. The percentage of treatment accomplishment was higher than the average in five regions (i.e., Kanto, Hokuriku, Tokai, Chugoku, and Kyushu) and lower than the average in the other four (*P* = 0.009) (Fig. 2). SVR, TVR, NVR, and undetermined response differed significantly among the nine regions (Fig. 3, *P* < 0.001). Such regional disparities were more clearly demonstrated by stratification with the HCV genotypes and viral loads. As shown in Fig. 4, regional disparities regarding treatment performance were detected in the genotype 2 subgroup (*P* = 0.018, with Bonferroni method), especially with high viral load (*P* = 0.036), but not in genotype 1. However, regional disparities regarding treatment outcome were confirmed in the subgroups with high viral load, regardless of HCV genotypes.

### Predictive factors for treatment performance

To determine the factors associated with treatment accomplishment with P/R, univariate analysis was performed (Table 2). Treatment accomplishment rate was significantly lower in the elderly group (77.2 vs. 85.1 % [<65 years]), IFN-experienced cases (81.7 vs. 83.1 % [IFN naive]), genotype 1 group (79.0 vs. 91.1 % [genotype 2 + 3]), and high viral load group (82.7 vs. 85.3 % [low viral load]). Higher levels of serum aspartate aminotransferase (AST), FIB-4 index, and lower platelet counts were significantly associated with treatment withdrawal. In addition, treatment accomplishment rate gradually decreased in patients whose treatment was initiated in 2009 and later (i.e., 85.2 % [-2008], 82.8 % [2009], 80.2 % [2010-]; *P* < 0.001).

**Table 2:** Factors associated with withdrawal of peginterferon-α and ribavirin treatment in chronic hepatitis C patients

		Groups		Univariate analysis		Multivariate analysis		
Factors		Treatment accomplishment	Treatment withdrawal	Coefficient	*P*-value	B	Odds ratio	95 % C.I.
Gender (Male/Female)		5824/5766	1184/1226	1.004	0.325	0.000	1.000	0.911–1.099
**Age** (≥65 years/<65 years)		3146/8423	930/1473	127.536	<0.001	−0.326	0.722	0.651–0.800
History of IFN treatment (naive/experienced)		8463/2928	1716/657	4.005	0.047	0.032	1.033	0.929–1.147
**Genotype** (1/2 + 3)		7326/4050	1953/397	311.818	<0.001	−1.007	0.365	0.324–0.412
Viral load (high/low)		10713/817	2248/141	4.327	0.038	−0.091	0.913	0.750–1.112
**Pre-AST** (U/L)^a^		43 [30–68] (n = 11511)	48 [34–73] (n = 2385)		<0.001	0.001	1.001	1.000–1.003
Pre-ALT (U/L)^a^		51 [31–87] (n = 11517)	52 [34–81] (n = 2384)		0.517			
Pre-PLT (x10^4^/μL)		16.0 [13.0–20.0]	15.0 [12.0–19.0]		<0.001	0.001	1.001	0.990–1.013
		(n = 11381)	(n = 2349)					
**FIB-4 index**		2.26 [1.48–3.42]	2.84 [1.84–4.28]		<0.001	−0.125	0.882	0.850–0.916
		(n = 11349)	(n = 2341)					
**Year of starting treatment**	– 2008	4624	804				Reference	
	2009	2845	588	45.561	<0.001	−0.219	0.803	0.710–0.909
	2010–	4119	1015			−0.496	0.609	0.545–0.680
**Region**	Hokkaido/Tohoku	1404	318			−0.242	0.785	0.666–0.925
	Kanto	793	124			0.180	1.197	0.958–1.497
	Shin-etsu	528	114			0.020	1.020	0.802–1.297
	Hokuriku	717	148			0.036	1.037	0.838–1.283
	Tokai	814	145	20.418	0.009	0.231	1.260	1.019–1.558
	Kinki	2385	544			−0.048	0.953	0.827–1.098
	Chugoku	1890	380			0.006	1.006	0.864–1.172
	Shikoku	476	115			−0.240	0.787	0.619–0.999
	Kyushu	2589	522				Reference	

The values of pre-AST, pre-ALT, pre-PLT, and FIB-4 index are shown as median [interquartile range]

^**a**^Since pre-AST and pre-ALT were closely correlated (r = 0.872; *P* < 0.001), only pre-AST was included in multivariate analysis. Significant factors by multivariate analysis are shown in bold. The patients whose treatment performance could not be determined were excluded from this analysis (*n* = 55)

*ALT*, Alanine aminotransferase; *AST*, Aspartate aminotransferase; *C.I.*, Confidence interval; *FIB-4*, Fibrosis-4; *IFN*, Interferon; *PLT*, Platelets; *SVR*, Sustained virological response

We found that treatment accomplishment rate differed among the nine regions in the entire P/R-treated cohort (*P* = 0.009, Fig. 2), especially in the genotype 2 subgroup with high viral load (Fig. 4, *upper panel*), and was closely associated with treatment outcome (i.e., 95.8, 84.8 and 45.8 % in SVR, TVR and NVR, respectively). According to the multivariate and simultaneous logistic regression analysis (Table 2), the following six factors independently contributed to treatment withdrawal: old age, genotype 1, high serum AST levels, high FIB-4 index, later year of starting treatment, and region. Treatment accomplishment rate was lower in Hokkaido/Tohoku and Shikoku, and higher in Tokai among 9 regions.

### Predictive factors for treatment outcome

To evaluate factors associated with SVR in P/R therapy, univariate analysis was performed (Table 3). The SVR rates were lower in female patients (54.8 vs. 63.6 % [male patients]), elderly patients (47.3 vs. 64.1 % [<65 years]), IFN-experienced cases (49.7 vs. 62.4 % [IFN-naive]), genotype 1 group (48.9 vs. 80.8 % [genotype 2 + 3]), and high viral load group (57.7 vs. 80.2 % [low viral load]). Serum AST and alanine aminotransferase (ALT) levels, peripheral platelet counts, and FIB-4 index were significantly different between SVR and non-SVR. In addition, SVR rates were increased in patients who had initiated treatment in 2009 and later (i.e., 56.8 % [-2008], 59.0 % [2009], and 60.6 % [2010-]; *P* = 0.019).

**Table 3:** Factors associated with non-sustained virological response to peginterferon-α and ribavirin treatment in chronic hepatitis C patients

		Groups		Univariate analysis		Multivariate analysis		
Factors		SVR	Non-SVR	Coefficient	*P*-value	B	Odds ratio	95 % C.I.
**Gender** (Male/Female)		4234/3740	2426/3080	106.402	<0.001	−0.302	0.739	0.683–0.800
**Age** (≥65 years/<65 years)		1865/6093	2079/3414	325.742	<0.001	−0.392	0.676	0.618–0.739
**History of IFN treatment** (naïve/experienced)		6106/1733	3676/1755	172.508	<0.001	−0.407	0.665	0.610–0.726
**Genotype** (1/2 + 3)		4386/3439	4577/818	1212.744	<0.001	−1.344	0.261	0.238–0.286
**Viral load** (high/low)		7204/736	5281/182	178.857	<0.001	−1.213	0.297	0.247–0.359
Pre-AST (U/L)^*^		43 [30–69] (n = 7928)	45 [32–68] (n = 5457)		<0.001			
**Pre-ALT** (U/L)^*^		54 [31–93] (n = 7931)	48 [31–75] (n = 5459)		<0.001	0.005	1.005	1.004–1.005
**Pre-PLT** (x10^4^/μL)		17.0 [14.0–21.0] (n = 7855)	15.0 [12.0–19.0] (n = 5374)		<0.001	0.018	1.019	1.009–1.029
**FIB-4 index**		2.11 [1.36–3.20] (n = 7833)	2.77 [1.86–4.15] (n = 5357)		<0.001	−0.164	0.849	0.821–0.877
Year of starting treatment	– 2008	3062	2327				Reference	
	2009	1958	1362	7.949	0.019	−0.085	0.918	0.831–1.015
	2010–	2950	1915			−0.095	0.909	0.830–0.997
**Region**	Hokkaido/Tohoku	941	711			−0.288	0.750	0.653–0.861
	Kanto	547	316			−0.064	0.938	0.786–1.119
	Shin-etsu	361	271			−0.069	0.933	0.765–1.137
	Hokuriku	490	354			−0.032	0.969	0.815–1.152
	Tokai	557	381	21.777	0.005	−0.069	0.934	0.789–1.105
	Kinki	1637	1160			−0.116	0.891	0.790–1.004
	Chugoku	1322	920			−0.044	0.957	0.844–1.085
	Shikoku	314	257			−0.399	0.671	0.546–0.825
	Kyushu	1808	1138				Reference	

The values of pre-AST, pre-ALT, pre-PLT, and FIB-4 index are shown as median [interquartile range]

^**a**^Since pre-AST and pre-ALT were closely correlated (r = 0.872; *P* < 0.001), only pre-ALT was included in multivariate analysis. Significant factors by multivariate analysis are shown in bold. The patients whose treatment outcome could not be determined were excluded from this analysis (n = 576)

*ALT*, Alanine aminotransferase; *AST*, Aspartate aminotransferase; *C.I.*, Confidence interval; *FIB-4*, Fibrosis-4; *IFN*, Interferon; *PLT*, Platelets; *SVR*, Sustained virological response

We were able to demonstrate that the treatment outcome was significantly different among the nine regions in the entire P/R-treated cohort (*P* < 0.001) and in the subgroups with high viral load, regardless of HCV genotypes (Fig. 4, *lower panel*). Furthermore, SVR rates were closely associated with treatment performance (i.e., 67.7 and 15.5 % in treatment accomplishment and withdrawal, respectively; data not shown). According to the multivariate and simultaneous logistic regression analysis (Table 3), most of the factors independently contributed to non-SVR (i.e., female sex, old age, experienced IFN treatment, genotype 1, high viral load, low serum ALT levels, low peripheral platelet counts, high FIB-4 index, and region). SVR rates in patients who had initiated treatment in 2010 and later were higher during the periods examined. In addition, SVR rates were significantly lower in Hokkaido/Tohoku and Shikoku among 9 regions.

## Discussion

In this study, regional disparities in the demographic features of IFN-treated patients (i.e., age, sex, history of IFN treatment, and prevalence of HCV genotypes) in Japan have been demonstrated for the first time. Furthermore, regional disparities in treatment accomplishment and outcome of standard treatment with P/R were also observed. Regional disparities and other known predictive factors were independently associated with treatment performance and outcome. The inconsistent increase in treatment withdrawal in patients who started treatment later in the year could be explained by the gradual spread of concepts related to response-guided therapy [16], which is chiefly based on cost effectiveness, especially in treatment of hard-to-cure patients [17].

It should be emphasized that the rates of treatment accomplishment and SVR in the Hokkaido/Tohoku and Shikoku regions were significantly lower among 9 regions. A strong correlation between SVR rate and treatment accomplishment rate (*r* = 0.879, *P* = 0.002; Additional file 1: Figure S1) suggests the presence of factors that influence treatment accomplishment, leading to non-SVR, in these two regions. Although the percentage of IFN-experienced cases in Shikoku was higher than in other regions, a history of IFN treatment was not chosen as a predictive factor for treatment accomplishment by multivariate analysis. The reasons for treatment withdrawal were divided into two categories: severe adverse events, and unrelated incidents. The proportions of these two categories were ~60 and ~40 %, respectively, throughout Japan, and there was no regional difference among the nine regions (Additional file 2: Figure S2). However, in the category of unrelated incidents, the proportion with “poor response to P/R, according to criteria for response-guided therapy” differed among the nine regions (*P* = 0.019; Additional file 3: Figure S3). In Hokkaido/Tohoku, the proportion was the lowest among the nine regions, which suggests the presence of the other factors peculiar to this region. Treatment accomplishment rate and SVR rate were not associated with the proportion of elderly patients in the P/R-treated cohort (Additional file 4: Figure S4 and Additional file 5: Figure S5) or the numbers of specialists in hepatology, designated by the Japan Society of Hepatology, per 100,000 people in each region (Additional file 6: Figure S6 and Additional file 7: Figure S7). Therefore, we need to consider another possibility such as limited accessibility to medical resources, particularly in the regions with low population density. Hokkaido and Tohoku are the regions with the lowest population density in Japan (Additional file 8: Figure S8). Therefore, consultation networks between base hospitals and local clinics should be constructed and properly managed by public health actions, especially in those regions.

Many findings similar to our study have recently been accumulated by public health policies regarding regional disparities in the treatment outcome of acute illness and malignant disorders. O’Connor et al. have reported that substantial geographic variation exists in the treatment of patients with acute myocardial infarction in the US, probably resulting from underuse of therapies with proven benefit in local clinical practices [18]. Gentry et al. recently proposed that geographic disparities in the 90-day transplant rates and waiting-list death rates for liver transplantation in the US could be reduced by redistricting based on optimal liver allocation [19]. Brantley-Sieders et al. also found that breast cancer mortality rates, which varied among counties in Middle Tennessee, correlated with additional risk factors (i.e., mammography screening and socioeconomic status) and proposed resources to reduce breast cancer mortality [20]. In contrast, information regarding regional disparities for the treatment of CHC with P/R is limited. In the IDEAL Study, no significant differences were detected in the various metrics of quality and site performance (i.e., adherence, adverse events, treatment withdrawal, on-treatment virological response, and SVR) between 76 academic-based and 42 community-based centers in the US [21]. Based on these previous studies, further evaluations of local clinical practices should be mandatory to explore the reasons for regional disparities in treatment and outcome of P/R therapy in Japan, especially in the two regions identified in our study, Hokkaido/Tohoku and Shikoku. Again, access to medical treatment would be a serious burden on patients who are company employees or residents in the regions with low population density, partly because the self-injection of peginterferon-α has not yet been approved by the Pharmaceutical Affairs Law in Japan.

Owing to the introductions of IFN-free regimens of DAAs, use of IFN is limited to settings in which new treatments may initially be too expensive to be utilized [22]. Considering that HCV infection may cause chronic and morbid liver diseases (i.e., liver cirrhosis or hepatocellular carcinoma), such a perspective is acceptable in general. However, at the same time, we should pay close attention to the presence or emergence of resistance to those DAAs. In particular, with the recently approved simeprevir, up to 40 % of patients in the US infected with genotype 1a HCV have Q80K mutation before treatment. Thus, it is strongly recommended this polymorphism should be screened prior to treatment with P/R plus simeprevir, according to the American Association for the Study of Liver Diseases/Infectious Diseases Society of America Recommendations [23]. In addition, we need to think about the possibility of unexpected emergence of resistance to various forthcoming DAAs, in case of patients with poor drug adherence or viral breakthrough. The necessity for adequate education of general physicians, as well as HCV-infected patients, should be emphasized, for standardized performance and outcome of the forthcoming treatment, including DAAs.

There were several limitations to this study. First, our nationwide database consisted of only ~20 % of patients in the prefectures who benefited from the governmental subsidy policy, and was not reflective of all patients in Japan. However, considering that the difference in the percentage of elderly patients (≥65 years) between our collected reports and all applicants for this subsidy during the initial 3 years in each prefecture was only 1.3 % (median; interquartile range:–2.7 to 4.1 %; preliminary analysis for 26 prefectures), we may assume that our database represents all the patients who benefited from this subsidy policy. Second, the number of collected parameters was inevitably influenced by the willingness of the doctors to complete the reports during daily clinical practice. Thereby, information regarding the doctors’ specialty, drug adherence, or treatment outcome of previously administered IFN could not be included in this analysis. Third, generalizability of our findings is necessary, however, it is difficult for this type of large cohort study for P/R therapy in a rapidly changing era of treatment modalities for CHC. Finally, we were unable to collect information regarding the accessibility to medical resources for individual patients, which may have affected treatment accomplishment. Further investigation would be indispensable to evaluate this issue, by analyzing additional factors in those areas, such as a going-to-hospital time, availability of consultation networks between base hospitals and local clinics, and so on.

## Conclusions

Treatment performance and outcome in patients with CHC are not yet standardized in Japan and further investigations to solve the problems of regional disparities should be performed from the viewpoint of local clinical practice. The policies for treatment of hepatitis by the Japanese government should be formulated to correspond with the characteristics of the respective jurisdictions so that the patients with viral hepatitis may receive the highest standard of medical care, regardless of the locality where they reside.

## Additional files

Additional file 1: Figure S1. Correlation between treatment accomplishment and sustained virological response (SVR) rates in patients treated by peginterferon-α and ribavirin in nine regions of Japan. SVR rates were strongly correlated with the rates of treatment accomplishment (*r* = 0.879, *P* = 0.002). Additional file 2: Figure S2. Rates of severe adverse events (SAE) and unrelated incidents among the reasons for withdrawal of peginterferon-α and ribavirin therapy. There was no regional difference among nine regions of Japan (*P* = 0.075). Additional file 3: Figure S3. Rates of poor response to peginterferon-α and ribavirin (P/R) and other agents as reasons for treatment withdrawal because of unrelated incidents. The proportion of patients with poor response to P/R, according to criteria for response-guided therapy differed among nine regions of Japan (*P* = 0.019). In Hokkaido/Tohoku the proportion was the lowest. Additional file 4: Figure S4. Proportions of elderly patients and treatment accomplishment rate for peginterferon-α and ribavirin in nine regions of Japan. No correlation was found between the proportions of elderly patients and treatment accomplishment rate (*r* = −0.107, *P* = 0.783). Additional file 5: Figure S5. Proportions of elderly patients and sustained virological response (SVR) rate in patients treated by peginterferon-α and ribavirin in nine regions of Japan. No correlation was found between the proportions of elderly patients and SVR rate (*r* = 0.133, *P* = 0.733). Additional file 6: Figure S6. Numbers of specialists in hepatology and treatment accomplishment rate in patients treated by peginterferon-α and ribavirin in nine regions of Japan. No correlation was found between these two parameters (*r* = 0.030, *P* = 0.939). Additional file 7: Figure S7. Numbers of specialists in hepatology and sustained virological response (SVR) rate in patients treated by peginterferon-α and ribavirin in nine regions of Japan. No correlation was found between these two parameters (*r* = 0.088, *P* = 0.822). Additional file 8: Figure S8. Population density in each region in Japan. The figures were calculated based on the Basic Resident Register of Japan in 2013 (http://www.soumu.go.jp/menu_news/s-news/01gyosei02_02000055.html). In this figure, data for the Hokkaido and Tohoku regions are shown separately.

## Abbreviations

ALT: Alanine aminotransferase
AST: Aspartate aminotransferase
CHC: Chronic hepatitis C
CI: Confidence interval
DAAs: Directly acting antivirals
FIB-4: Fibrosis-4
HBV: Hepatitis B virus
HCV: Hepatitis C virus
IFN: Interferon
NVR: Nonvirological response
PLT: Platelets
P/R: Peginterferon-α and ribavirin
SVR: Sustained virological response
TVR: Transient virological response
