# Supplementary material for: Regional disparities in interferon therapy for chronic hepatitis C in Japan: a nationwide retrospective cohort study
Source: BMC Public Health. 2015 Jun 19;15:566. doi: 10.1186/s12889-015-1891-2 (PMC4474553; doi:10.1186/s12889-015-1891-2)
Supplement: Additional file 1: Figure S1. — Correlation between treatment accomplishment and sustained virological response (SVR) rates in patients treated by peginterferon-α and ribavirin in nine regions of Japan. SVR rates were strongly correlated with the rates of treatment accomplishment (r = 0.879, P = 0.002). [file 12889_2015_1891_MOESM1_ESM.pdf]

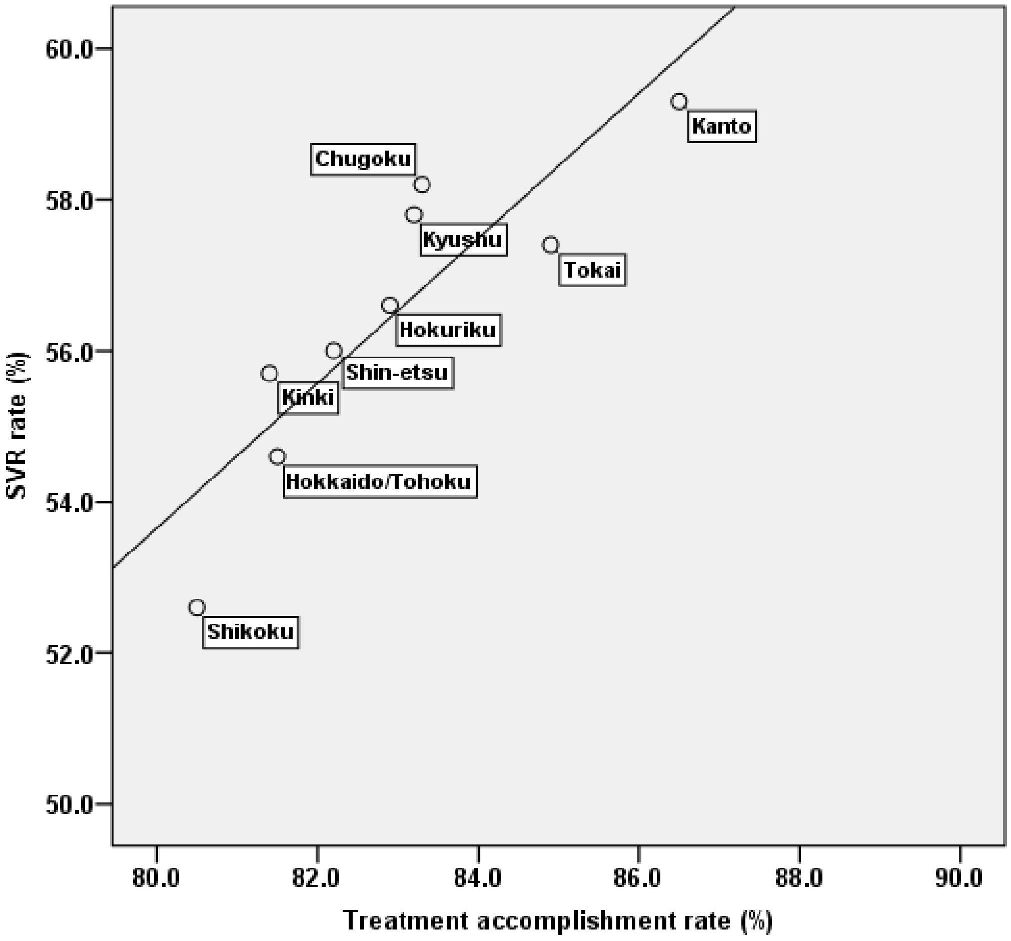


**Additional Figure 1. Correlation between treatment accomplishment and sustained virological response (SVR) rates in patients treated by peginterferon-α and ribavirin in nine regions of Japan.** SVR rates were strongly correlated with the rates of treatment accomplishment ($r = 0.879$, $P = 0.002$).
