# Supplementary material for: Regional disparities in interferon therapy for chronic hepatitis C in Japan: a nationwide retrospective cohort study
Source: BMC Public Health. 2015 Jun 19;15:566. doi: 10.1186/s12889-015-1891-2 (PMC4474553; doi:10.1186/s12889-015-1891-2)
Supplement: Additional file 2: Figure S2. — Rates of severe adverse events (SAE) and unrelated incidents among the reasons for withdrawal of peginterferon-α and ribavirin therapy. There was no regional difference among nine regions of Japan (P = 0.075). [file 12889_2015_1891_MOESM2_ESM.pdf]

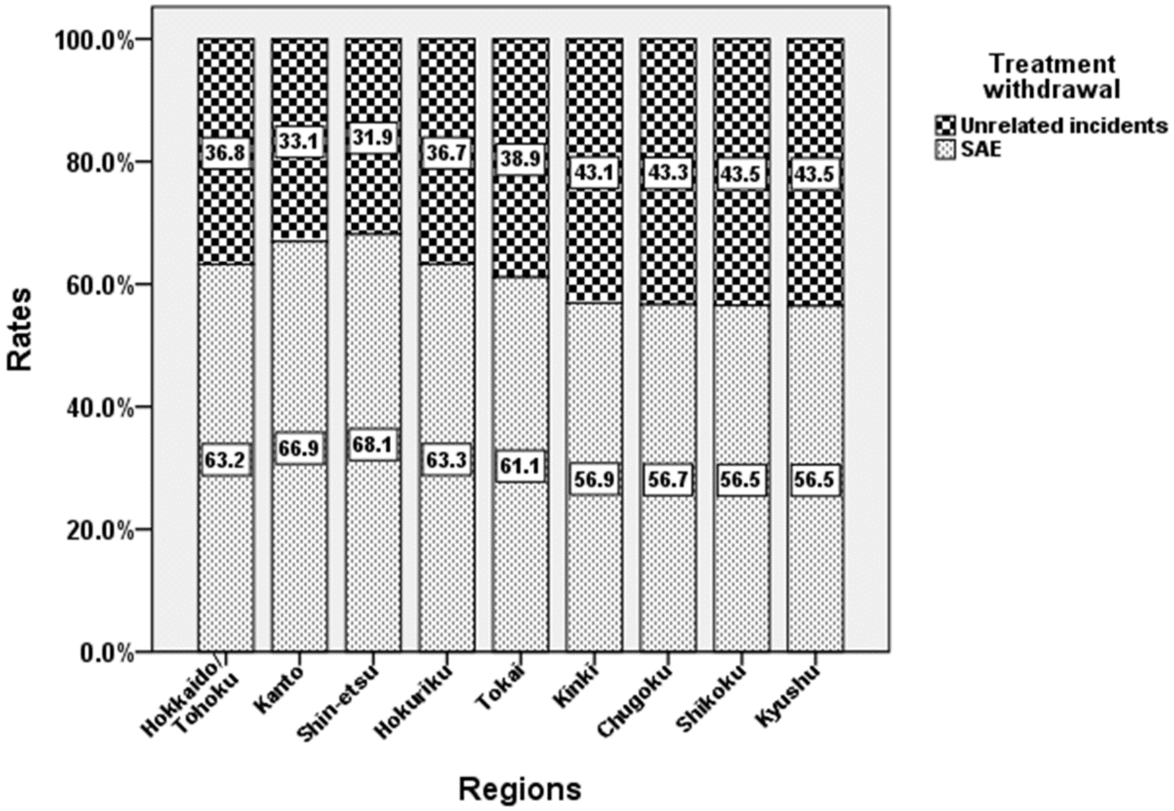

Additional Figure 2. Rates of severe adverse events (SAE) and unrelated incidents among the reasons for withdrawal of peginterferon-α and ribavirin therapy. There was no regional difference among nine regions of Japan (*P* = 0.075).
