# Supplementary material for: Regional disparities in interferon therapy for chronic hepatitis C in Japan: a nationwide retrospective cohort study
Source: BMC Public Health. 2015 Jun 19;15:566. doi: 10.1186/s12889-015-1891-2 (PMC4474553; doi:10.1186/s12889-015-1891-2)
Supplement: Additional file 3: Figure S3. — Rates of poor response to peginterferon-α and ribavirin (P/R) and other agents as reasons for treatment withdrawal because of unrelated incidents. The proportion of patients with poor response to P/R, according to criteria for response-guided therapy differed among nine regions of Japan (P = 0.019). In Hokkaido/Tohoku the proportion was the lowest. [file 12889_2015_1891_MOESM3_ESM.pdf]

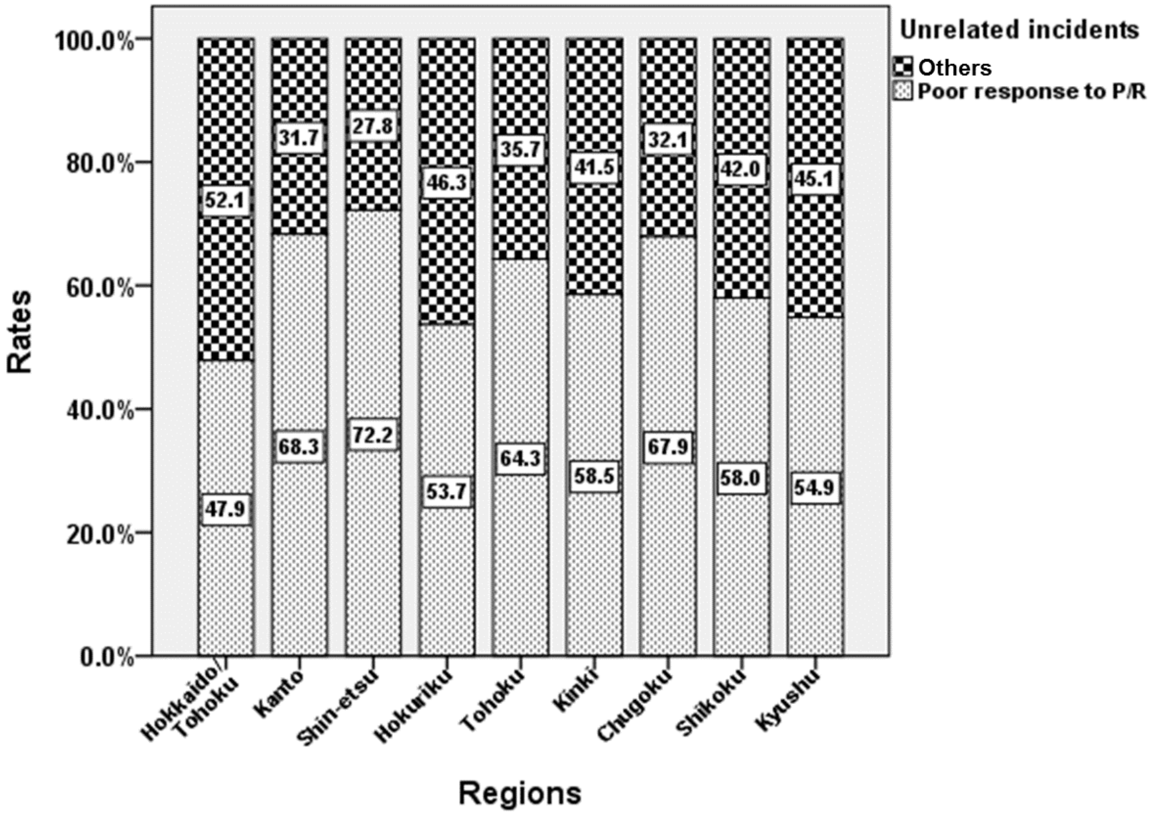

Additional Figure 3. Rates of poor response to peginterferon-α and ribavirin (P/R) and other agents as reasons for treatment withdrawal because of unrelated incidents. The proportion of patients with poor response to P/R, according to criteria for response-guided therapy differed among nine regions of Japan (P = 0.019). In Hokkaido/Tohoku the proportion was the lowest.
