# Supplementary material for: Regional disparities in interferon therapy for chronic hepatitis C in Japan: a nationwide retrospective cohort study
Source: BMC Public Health. 2015 Jun 19;15:566. doi: 10.1186/s12889-015-1891-2 (PMC4474553; doi:10.1186/s12889-015-1891-2)
Supplement: Additional file 4: Figure S4. — Proportions of elderly patients and treatment accomplishment rate for peginterferon-α and ribavirin in nine regions of Japan. No correlation was found between the proportions of elderly patients and treatment accomplishment rate (r = −0.107, P = 0.783). [file 12889_2015_1891_MOESM4_ESM.pdf]

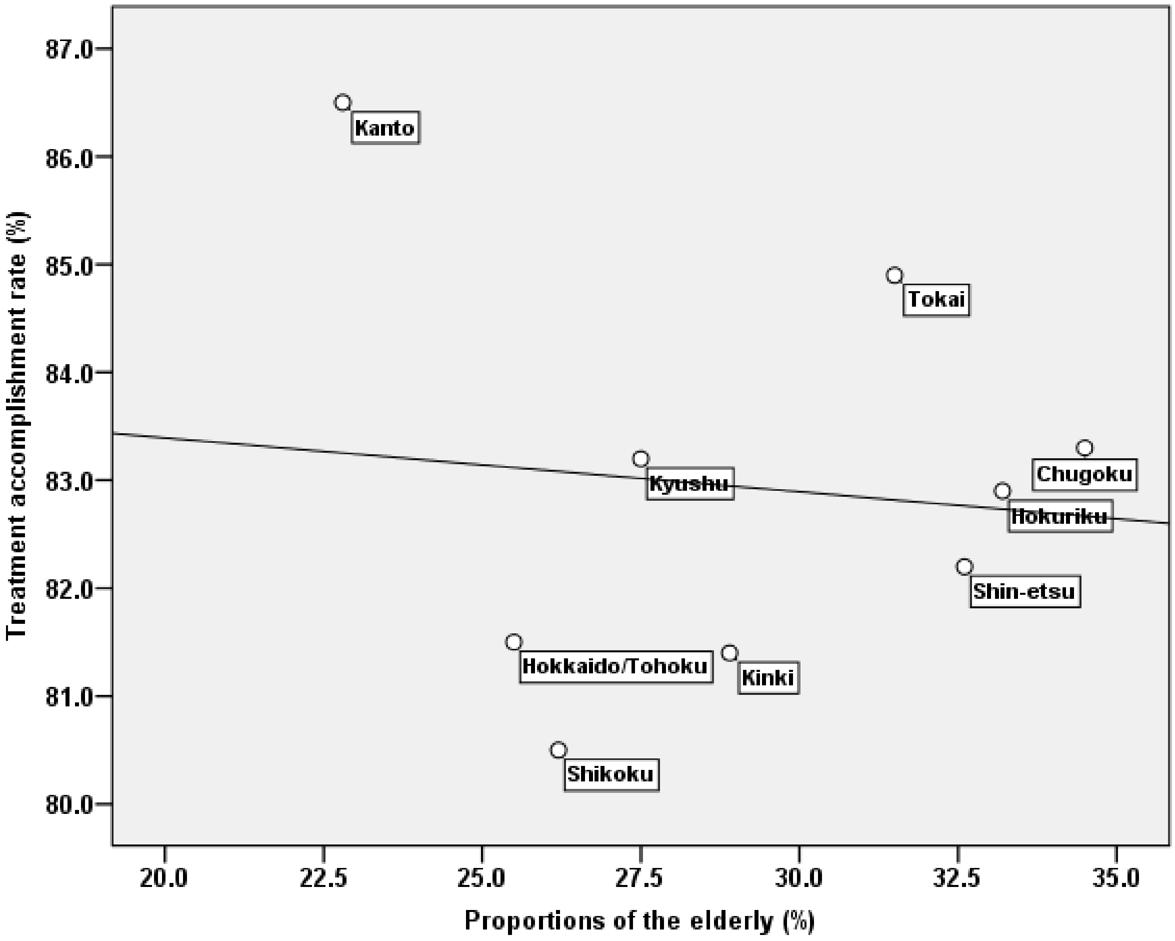

Additional Figure 4. Proportions of elderly patients and treatment accomplishment rate for peginterferon-α and ribavirin in nine regions of Japan. No correlation was found between the proportions of elderly patients and treatment accomplishment rate (r = –0.107, P = 0.783).
