# Supplementary material for: Regional disparities in interferon therapy for chronic hepatitis C in Japan: a nationwide retrospective cohort study
Source: BMC Public Health. 2015 Jun 19;15:566. doi: 10.1186/s12889-015-1891-2 (PMC4474553; doi:10.1186/s12889-015-1891-2)
Supplement: Additional file 5: Figure S5. — Proportions of elderly patients and sustained virological response (SVR) rate in patients treated by peginterferon-α and ribavirin in nine regions of Japan. No correlation was found between the proportions of elderly patients and SVR rate (r = 0.133, P = 0.733). [file 12889_2015_1891_MOESM5_ESM.pdf]

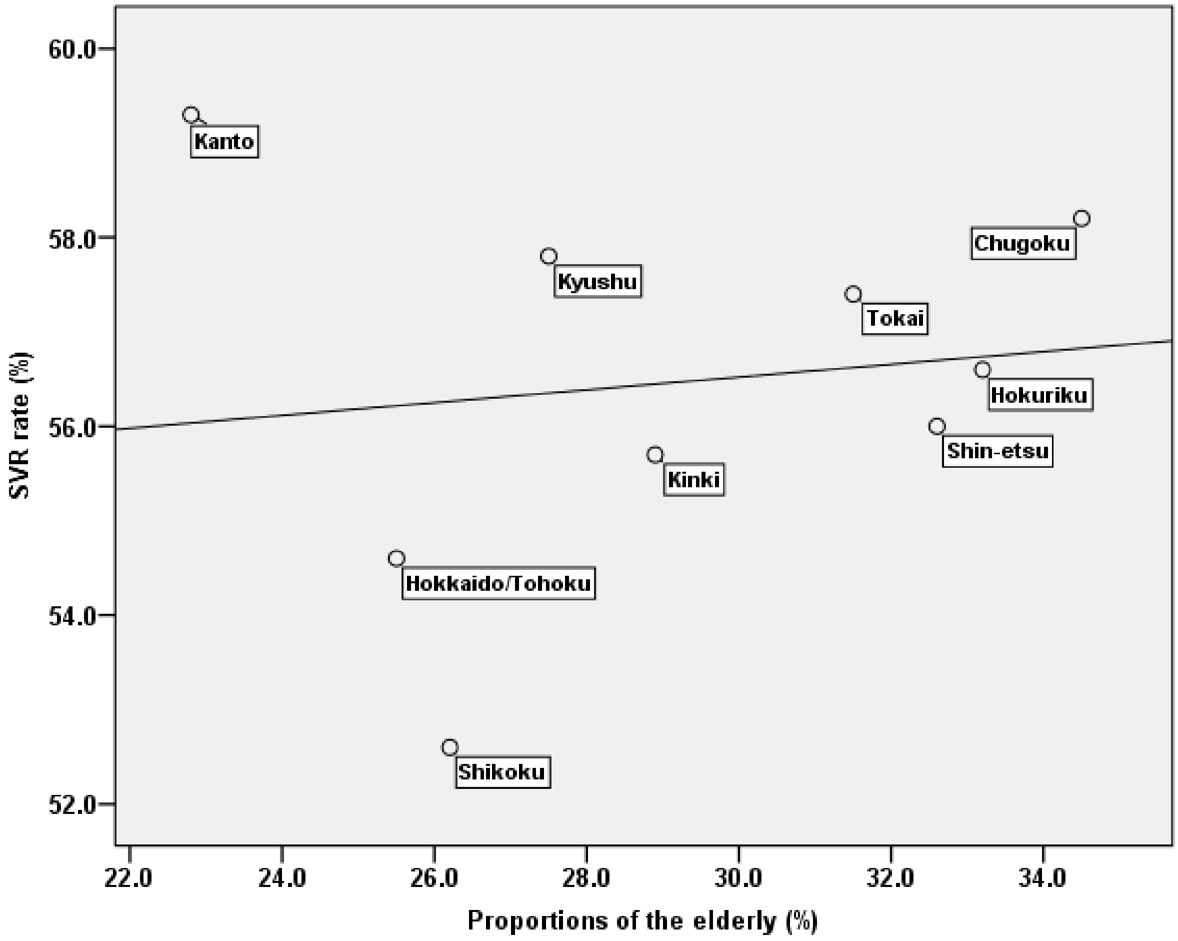


**Additional Figure 5. Proportions of elderly patients and sustained virological response (SVR) rate in patients treated by peginterferon-α and ribavirin in nine regions of Japan.** No correlation was found between the proportions of elderly patients and SVR rate ($r$ = 0.133, $P$ = 0.733).
