# Supplementary material for: Regional disparities in interferon therapy for chronic hepatitis C in Japan: a nationwide retrospective cohort study
Source: BMC Public Health. 2015 Jun 19;15:566. doi: 10.1186/s12889-015-1891-2 (PMC4474553; doi:10.1186/s12889-015-1891-2)
Supplement: Additional file 7: Figure S7. — Numbers of specialists in hepatology and sustained virological response (SVR) rate in patients treated by peginterferon-α and ribavirin in nine regions of Japan. No correlation was found between these two parameters (r = 0.088, P = 0.822). [file 12889_2015_1891_MOESM7_ESM.pdf]

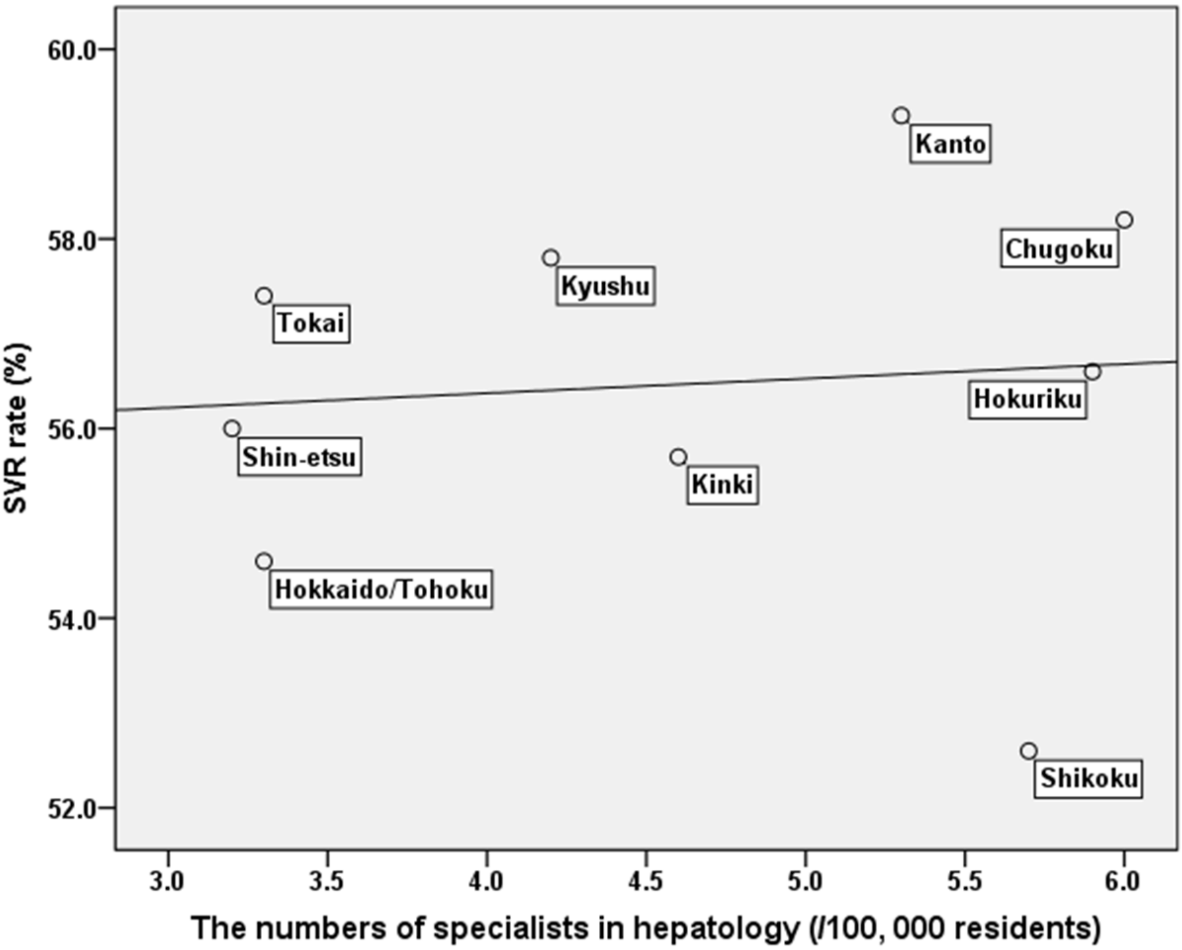

Additional Figure 7. Numbers of specialists in hepatology and sustained virological response (SVR) rate in patients treated by peginterferon-α and ribavirin in nine regions of Japan. No correlation was found between these two parameters (r = 0.088, P = 0.822).
