# Supplementary material for: Regional disparities in interferon therapy for chronic hepatitis C in Japan: a nationwide retrospective cohort study
Source: BMC Public Health. 2015 Jun 19;15:566. doi: 10.1186/s12889-015-1891-2 (PMC4474553; doi:10.1186/s12889-015-1891-2)
Supplement: Additional file 8: Figure S8. — Population density in each region in Japan. The figures were calculated based on the Basic Resident Register of Japan in 2013 (http://www.soumu.go.jp/menu_news/s-news/01gyosei02_02000055.html). In this figure, data for the Hokkaido and Tohoku regions are shown separately. [file 12889_2015_1891_MOESM8_ESM.pdf]

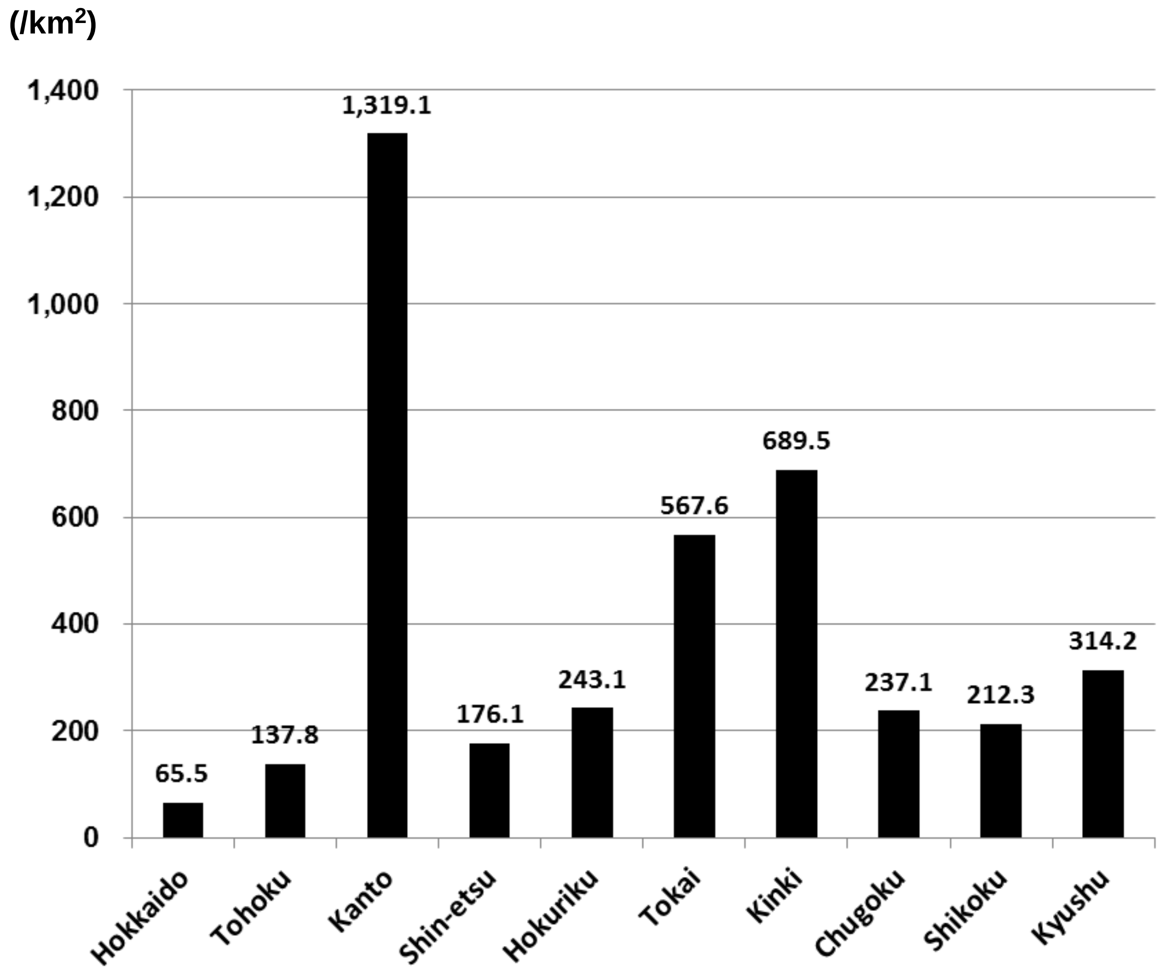

Additional Figure 8. Population density in each region in Japan. The figures were calculated based on the Basic Resident Register of Japan in 2013 (http://www.soumu.go.jp/menu_news/s-news/01gyosei02_02000055.html). In this figure, data for the Hokkaido and Tohoku regions are shown separately.
